# Collagenase-based wound debridement agent induces extracellular matrix supporting phenotype in macrophages

**DOI:** 10.1038/s41598-024-53424-2

**Published:** 2024-02-08

**Authors:** Pradipta Banerjee, Amitava Das, Kanhaiya Singh, Savita Khanna, Chandan K. Sen, Sashwati Roy

**Affiliations:** 1 Department of Surgery, McGowan Institute for Regenerative Medicine, University of Pittsburgh School of Medicine, 450 Technology Drive, Room#421, Pittsburgh, PA 15219 USA; 2 Indiana Center for Regenerative Medicine and Engineering, Indiana University School of Medicine, Indianapolis, IN USA

**Keywords:** Cell biology, Immunology, Molecular biology, Physiology

## Abstract

Macrophages assume diverse phenotypes and functions in response to cues from the microenvironment. Earlier we reported an anti-inflammatory effect of Collagenase Santyl^®^ Ointment (CSO) and the active constituent of CSO (CS-API) on wound macrophages in resolving wound inflammation indicating roles beyond debridement in wound healing. Building upon our prior finding, this study aimed to understand the phenotypes and subsets of macrophages following treatment with CS-API. scRNA-sequencing was performed on human blood monocyte-derived macrophages (MDM) following treatment with CS-API for 24 h. Unbiased data analysis resulted in the identification of discrete macrophage subsets based on their gene expression profiles. Following CS-API treatment, clusters 3 and 4 displayed enrichment of macrophages with high expression of genes supporting extracellular matrix (ECM) function. IPA analysis identified the TGFβ-1 pathway as a key hub for the CS-API-mediated ECM-supportive phenotype of macrophages. Earlier we reported the physiological conversion of wound-site macrophages to fibroblasts in granulation tissue and impairment of such response in diabetic wounds, leading to compromised ECM and tensile strength. The findings that CSO can augment the physiological conversion of macrophages to fibroblast-like cells carry significant clinical implications. This existing clinical intervention, already employed for wound care, can be readily repurposed to improve the ECM response in chronic wounds.

## Introduction

Wound healing is a complex process that involves the coordinated interplay of various cellular and molecular events. The wounds that fail to progress through the normal stages of healing within an expected timeframe (typically > 30 days per CMS^1^) are known as chronic wounds. The economic impact of the care of chronic wounds encompasses various factors, including direct and indirect medical costs, and the overall societal burden. Per Medicare, the projected cost for management of chronic wounds in 2014 ranged from $28.1 to $96.8 billion in the US alone^2^. Adequate wound bed preparation is a crucial step in wound care that focuses on creating an optimal environment for the healing of chronic wounds^3^. One critical component of wound bed preparation is the removal of dead and necrotic tissues, the process also known as debridement^3,4^. Santyl^®^ or CSO (Collagenase Santyl^®^ Ointment) is an FDA-approved clostridial collagenase-based prescription ointment for effective enzymatic debridement of chronic wounds^5^. Expression of collagenases, a class of metalloproteinases, is required for wound healing, however, long-term, and the continued presence of these metalloproteinases at the wound site may adversely affect the healing process^6,7^. Elevated and prolonged expression of proteases produced during the inflammatory phase of healing can lead to excessive ECM degradation associated with impaired healing^6^. Chronic wounds exhibit heightened levels of MMPs alongside diminished tissue inhibitors of MMP (TIMP). This imbalance likely contributes to the hindered healing processes in these wounds. The MMP-1/TIMP-1 ratio has been proposed as a predictive marker for wound healing in diabetic foot ulcers^8^. We and others have proposed roles of CSO at the wound site that are beyond its role in debridement^9–12^.

Persistent non-resolving inflammation is a characteristic of chronic wounds^13,14^. Macrophages are critical cells that shape the inflammatory response at the wound-site^15,16^. Genetic approaches for selective depletion of macrophages demonstrated defective inflammatory, epithelialization, and granulation tissue responses^17,18^. Major functions of macrophages in the process of wound healing involve host-defense activities, mounting and resolution of the inflammatory response, removal of dead cells and debris, and supporting key wound healing processes including epithelialization, angiogenesis, and tissue remodeling^19^. A central role of macrophages in orchestrating ECM production and homeostasis during the injury repair process has been recognized^17,20^. The evidence supports that dynamic interaction between macrophages, and ECM components enhances the overall quality of tissue repair leading to an improved wound tensile strength^18,21^. Development of the significance of macrophages in ECM regulation rests on an appreciation that the production of appropriate ECM represents a major cornerstone of the healing process^22^.

Plasticity and heterogeneity are major characteristics of cells of myeloid lineages. Macrophages assume diverse phenotypes and functions in response to cues from microenvironment^23^. Recent scRNA-seq analysis studies have allowed for the identification of distinct macrophage subsets based on their gene expression profiles^23^. In our previous study, we first highlighted an anti-inflammatory effect of CSO specifically the active constituent of CSO, henceforth called CS-API on wound macrophages in resolving wound inflammation via STAT6 and NF-κB pathways^12^. Building upon our prior findings on unique responses of CSO on macrophage phenotype and function, the aim of this study was to utilize scRNA-seq technology to further understand the phenotypes, and subsets of macrophages following treatment with CSO. These studies unraveled unique functional pathways associated with CSO-induced macrophage subsets with significance in ECM regulation and wound healing.

## Results

**Figure 1:**
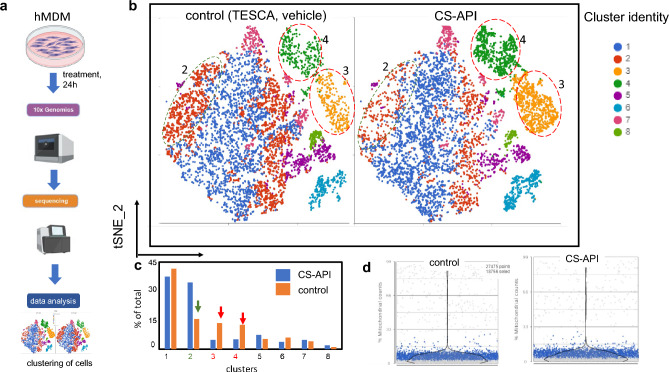
Single-cell RNA sequencing (scRNA seq) data analysis identifies 3 discrete MDM sub populations that are responsive to CS-API treatment. Human blood monocyte derived macrophages (hMDM) were treated with CS-API (250 ng/ml, 24 h). The cells were harvested and processed for scRNA seq analysis. Figure 1. (**a**) Schematic diagram illustrating the treatment of human monocyte-derived macrophages (hMDMs) and the workflow for obtaining and analyzing scRNA-seq data from hMDMs. (**b**) T-distributed stochastic neighbor embedding (tSNE) plots representing the results of scRNA-seq analysis on hMDM cells treated with CS-API or vehicle for 24 h. A total of eight major clusters (clusters 1–8) were identified and are depicted with distinct colors. The initial dataset consisted of 18,545 cells that passed the quality control criteria for further downstream analysis. (**c**) Bar graph displaying the percentage of total cells in each cluster. Clusters 2, 3, and 4 were found to be differentially expressed in the CS-API treated group. (**d**) Violin plot depicting the fraction of mitochondrial counts per cell in hMDMs treated with CS-API or vehicle control after filtration. Each data point represents a single cell, while the violin plot summarizes the distribution of the data.

Single-cell RNA sequencing (scRNA-seq) experiment investigating the effects of CS-API treatment on hMDM was performed to determine changes in the macrophage subset population in response to the treatment (Fig. 1). The schema of the experiment is presented (Fig. 1a). A total of 18,545 cells that passed the quality control parameters were subjected to downstream analyses (see “Methods”, Fig. 1d). Unsupervised clustering of single cells from the combined samples resulted in identification of 8 distinct clusters within hMDMs (Fig. 1b-c). Further analysis identified three transcriptionally distinct subpopulations (clusters 2, 3 and 4) that were either enriched (clusters 3 & 4) or diminished (cluster 2) in response to CS-API treatment (Fig. 1b) suggesting a unique macrophage subpopulation-specific response of CS-API. The total number of cells in the overall population of MDM subjected to scRNA analysis in CS-API or vehicle-treated were comparable. These findings provided insights into the transcriptional heterogeneity and differential gene expression patterns in hMDMs following treatment with CS-API, highlighting the potential impact of CS-API on macrophage phenotypes and cellular responses.

**Figure 2:**
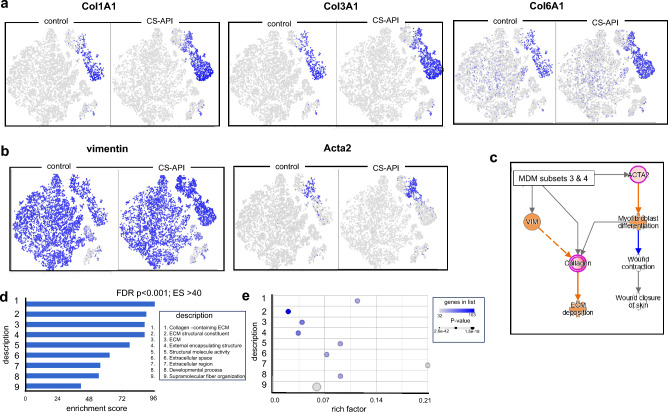
Identification of collagen producing macrophage subtypes following CS-API treatment. (**a**) t-SNE plots depicting the results of scRNA-seq analysis on human monocyte-derived macrophage (hMDM) cells treated with CS-API or vehicle for 24 h. The analysis revealed the presence of distinct macrophage subtypes, specifically clusters 3 and 4, which showed high expression levels of collagen types 1A1, 3A1, and 6A1. The cells expressing these transcripts are shown by blue colors in the corresponding plots. (**b**) In addition to increased collagen expression, the identified macrophage subtypes (clusters 3 and 4) also exhibited enhanced expression of vimentin (*VIM*) and *ACTA2*. (**c**) Ingenuity Pathway Analysis (IPA) was performed to assess the functional significance of the identified macrophage subtypes. The analysis revealed the significance of these clusters in extracellular matrix (ECM) deposition and wound healing. (**d**, **e**) Gene set enrichment analysis (GSEA), (**d**) the bar plot shows the enrichment scores of the significant (*p* < 0.0001) top 10 enrichment pathways; (**e**) bubble plot showing rich factor, p values and number of genes in the list.

The scRNA-seq analysis allowed for the identification of discrete macrophage subsets (clusters 2–4) based on their gene expression profiles. Notably, macrophages in clusters 3 and 4 displayed enrichment of macrophages expressing elevated levels of gene expression associated with ECM following CS-API treatment (Fig. 2). Several collagen-related genes, including *COL1A1, COL3A1, and COL6A1*, showed increased expression levels in the macrophages treated with CS-API compared to their control (TESCA) counterparts (Fig. 2a). In addition to increased collagen expression, the identified macrophage subtypes (clusters 3 and 4) also exhibited enhanced expression of vimentin (*VIM*) and *ACTA2* (Fig. 2b). These markers are associated with a more fibroblast-like phenotype. We and others have reported transition of macrophages to fibroblast-like cell^22,24,25^.Ingenuity Pathway Analysis (IPA) was performed to assess the functional significance of the identified macrophage subtypes (Fig. 2c). The analysis revealed the significance of these clusters in collagen production, ECM remodeling, and wound healing pathways. Gene Set Enrichment Analysis (GSEA) displayed ECM-related terms were highly significant and among top ten enriched terms (Fig. 2d, e).

**Figure 3:**
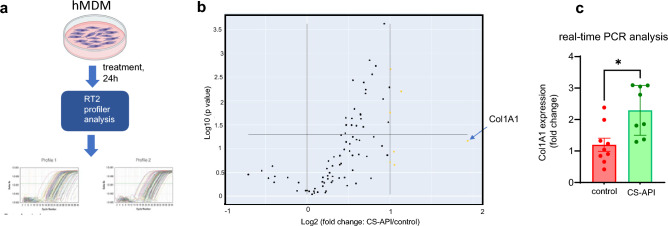
Volcano plot analysis of RT2 Profiler & RTPCR data reveals upregulation of collagen subtypes following CS-API treatment. (**a**) Schematic diagram of the validation of scRNA seq observation of Col1A1 expressing macrophage subtype using RT2 profiler and real time PCR analysis. (**b**) The volcano plot displays the differential gene expression analysis results of RT2 Profiler data, comparing CS-API-treated samples to control samples. Each data point represents a transcript, with the x-axis representing the log2 fold change and the y-axis representing the statistical significance (negative log10 p-value) of differential expression. The yellow data points indicate transcripts with a significant upregulation following CS-API treatment. Among the differentially expressed transcripts, *COL1A1* stands out as one of the most abundantly upregulated transcripts. (**c**) Independent RT-PCR analysis was performed to validate the upregulation of *COL1A1* mRNA following CS-API treatment. Expression levels of Col1A1 were measured using specific primers, and the results confirmed a significant increase in mRNA expression compared to control (TESCA buffer, vehicle) samples. Data presented as mean ± SEM (n = 6). *p < 0.05 compared to vehicle (control) treated MDM.

To verify the findings from scRNA analysis, RT2 profiler studies were performed using ECM-focused arrays (Fig. 3a). Using the RT2 Profiler data, a volcano plot analysis was performed to identify differentially expressed transcripts following CS-API treatment (Fig. 3b). The volcano plot revealed significant changes in gene expression, with *COL1A1* emerging as one of the most abundantly upregulated transcripts. *Col1A1* showed a significant increase in expression levels, indicating its potential role in the response to CS-API treatment and validating the findings from the scRNA seq analysis. Independent RT-PCR analysis was performed to assess the expression levels of *COL1A1* mRNA in samples treated with CS-API compared to vehicle control. The RT-PCR results confirmed a robust and significant upregulation of *COL1A1* mRNA expression following CS-API treatment, consistent with the findings from scRNA seq and RT2 profiler analysis (Fig. 3a-c).

**Figure 4:**
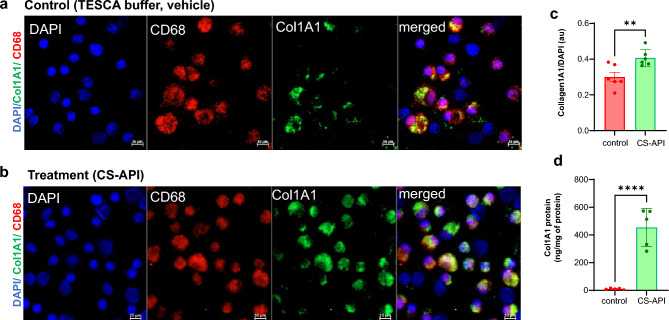
Independent immunohistochemistry validation of Col1A1 protein expression. (**a-c**) The immunocytochemistry (IHC) analysis involved the detection of Col1A1 protein using specific antibodies (Col1A1, green and CD68, red, marker for mature macrophages) using fluorescence microscopy. (**a**, **b**) microscopy images of MDM cells treated with either (**a**), control buffer (vehicle, TESCA); or (**b**), CS-API; (**c**) The bar graph presents the quantification of the IHC images. Scale bar = 10 mm. au = arbitrary units. (**d**) Col1A1 protein expression was determined by enzyme-linked immunosorbent assay (ELISA). Data are mean ± SEM from 3–5 independent IHC images in each group. *p < 0.05.

In addition to mRNA expression analysis, immunocytochemistry was employed to evaluate the protein expression of Col1A1 (Fig. 4a-c). The immunocytochemistry analysis involved the detection of Col1A1 protein using specific antibodies (Col1A1, green and CD68, red, marker for mature macrophages) in CS-API-treated and control (TESCA buffer, vehicle)-treated samples. The results demonstrated a marked increase in Col1A1 protein expression in cells treated with CS-API, confirming the upregulation observed at the transcript level. The enzyme-linked immunosorbent assay (ELISA) indicated the presence of significantly higher Col1A1 protein expression, post-CS-API treatment (Fig. 4d).

Collectively, the volcano plot analysis of RT2 Profiler data identified *COL1A1* as one of the most abundant transcripts upregulated following CS-API treatment. The independent validation through RT-PCR and immunocytochemistry analyses further confirmed the increased expression of *COL1A1* at both the mRNA and protein levels. These findings suggest a potential role for Col1A1 in the cellular response to CS-API treatment and highlight its significance in the context of CS-API-induced effects in wounds that are beyond its role in debridement.

**Figure 5:**
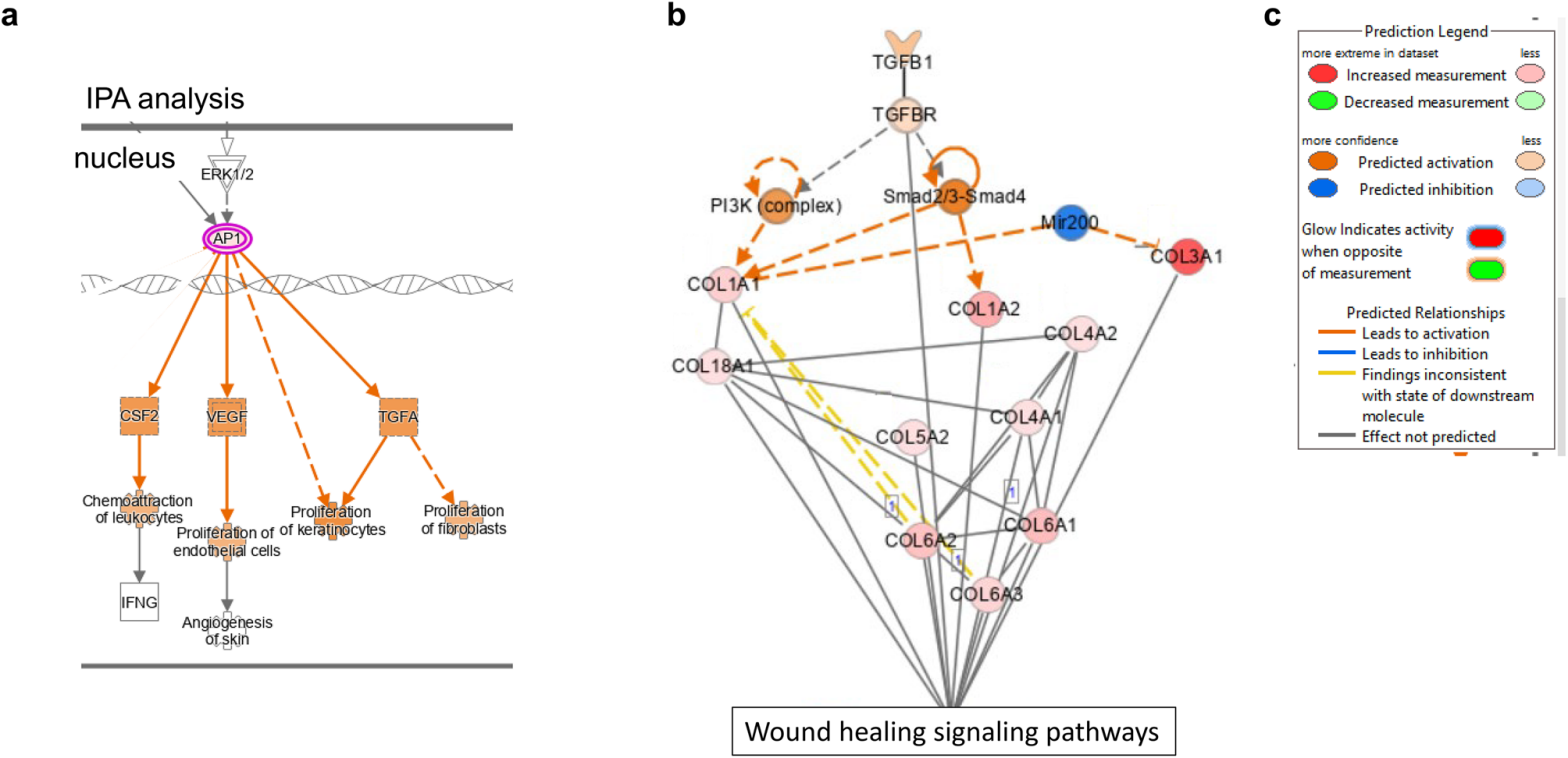
IPA Analysis of Single-cell RNA sequencing (scRNA-seq) Data. The IPA analysis was performed on scRNA-seq data from human monocyte-derived macrophages (hMDMs) following treatment with CS-API. (**a**) Analysis of wound healing related pathways identified upregulation of TGFβ-1, VEGF and CSF2 implicated in ECM generation, angiogenesis and proliferation and differentiation of myeloid cells, respectively. (**b**) Network analysis for upstream regulators of multiple collagen types upregulated in response to CS-API revealed TGFβ-1 and miR200b as hubs. (**c**) Prediction legend for the pathways presented in panels (**a**) and (**b**).

To determine the overall functional significance of the scRNA-seq data on macrophage responses to CS-API, IPA analysis was performed on scRNA-seq data from human monocyte-derived macrophages (hMDMs) following treatment with CS-API (Fig. 5). The analysis aimed at identifying the predicted pathways implicated in wound healing that were upregulated in hMDMs following CS-API treatment, the key predicted pathways upregulated included TGFβ-1, VEGF, and CSF2 pathways implicated in immune response, angiogenesis and ECM generation and homeostasis (Fig. 5a) providing insights into their functional implications in the wound healing response. Upstream analysis of collagen subtype response indicated the TGFβ-1 pathway as a key hub (Fig. 5b-c), revealing underlying molecular mechanisms of robust collagen response following CS-API treatment.

## Discussion

Wound healing is a complex process that involves the coordinated interplay of various cellular and molecular events^2,14,26^. Chronic wounds often exhibit compromised tensile strength, which can contribute to poor healing outcomes, increased risk of wound dehiscence, and overall impaired wound function. Several factors can contribute to compromised tensile strength in chronic wounds including prolonged inflammation, excessive production of MMPs, persistent infection, use of medications such as corticosteroids, and underlying conditions such as poor perfusion^27^. Adequate ECM dynamics and gain of tensile strength are essential for successful wound healing^28^. Pro-ECM interventions to improve the strength of chronic wounds are highly desirable. This study provided initial evidence that CSO, a clinically approved wound debridement agent has the potential to improve wound ECM response by converting macrophages to a pro-ECM fibroblast-like response. CSO is unique and one of the very few FDA-approved enzymatic debridement agents with biological activity for clinical application^29^.

Macrophage response is necessary for achieving normal repair processes^17,18^. Nonspecific inhibition of macrophages is detrimental to early matrix formation and wound strength^30^. While the classically activated M1 subtype and the alternatively activated M2 macrophages is a commonly used classification^31^, with the advent of scRNA seq studies it is recognized that this classification is an oversimplification of the macrophage subtypes involved in tissue repair and regeneration process^23,32^. As the wound progresses through the healing cascade, macrophages adopt distinct phenotypes to support healing including regulation of inflammatory response, angiogenesis, tissue regeneration, and extracellular matrix (ECM) deposition^22,23^. Initially, at the site of injury, macrophages exhibit a pro-inflammatory phenotype, secreting cytokines and chemokines that recruit endothelial cells fibroblasts, and myofibroblasts. Subsequently, macrophages transition to an anti-inflammatory phenotype, characterized by the secretion of anti-inflammatory cytokines, growth factors, and matrix metalloproteinases (MMPs)^23^. This study supports our previous research on the ability of CSO to transform macrophages into an anti-inflammatory phenotype^23^.

Macrophages display remarkable plasticity by converting into various cell types, such as endothelial cells, white adipocytes, and osteoclasts^22^. In an earlier study we provided definitive evidence, through lineage tracing and mechanistic studies, that at an early phase, the wound-site macrophages are the primary source of wound fibroblasts in the granulation tissue^22^. This concept has been independently verified by Guerrero-Juarez, et al., as they identified one subset of fibroblasts expressing hematopoietic markers, suggesting their myeloid origin^33^. Recently a fibrogenic macrophage subset was identified that is induced by type 3 inflammation^34^^.^ These macrophage-converted fibroblasts not only contribute to advancing the wound healing process beyond the inflammatory phase but also play a role in tissue remodeling after wound closure^22^. The findings from the above studies shed light on the significance of physiologically macrophage-converted fibroblast-like cells and their role in tissue repair, offering new perspectives on the acute inflammatory phase and the fate of macrophages at the injury site^22^. In the current study, the pro-ECM fibroblast-like phenotype observed via scRNA seq study indicates that CSO, through CS-API can augment the physiologic conversion of macrophages to fibroblast-like cells which may not only facilitate resolution of inflammation but also promote better granulation tissue formation. This finding has high translational significance in chronic wounds where conversion of wound macrophages into fibroblasts was noted to be impaired in diabetic individuals, leading to prolonged retention of macrophages in their pro-inflammatory state^22^. This finding from the current study along with our earlier report of promotion of an anti-inflammatory (M2) phenotype by CSO in diabetic wounds^12^ indicates a specific effect of CSO in modulating wound macrophage response to facilitate inflammation resolution, wound ECM deposition, and organization.

Type I collagen is a major component of the extracellular matrix (ECM) and is crucial for tissue structure and integrity^35,36^. While *COL1A1* expression is traditionally associated with fibroblasts and other mesenchymal cells, emerging evidence suggests that macrophages can also express *COL1A1* under certain conditions^22,37^. Macrophages with *COL1A1* expression can directly contribute to the accumulation of type I collagen in the ECM, thereby influencing tissue remodeling processes^22,35^. Growth factors transforming growth factor-beta (TGFβ) is known to regulate *COL1A1* expression via the cooperation of Sp1 and Smad proteins^38,39^. Ablation of macrophages in the wound using genetic approaches has been shown to consequently result in decreased expression of TGFβ, reduced proliferation of fibroblasts, and less ECM deposition^18^. IPA analysis in the current study predicted involvement of the TGFβ-1 pathway in the activation of the collagen subtype expression. The presence of macrophages expressing *COL1A1* following CS-API treatment highlights their potential role in ECM remodeling and granulation tissue maturation processes. The precise mechanisms governing *COL1A1* expression in macrophages are still under investigation. Further research is needed to fully understand the functional implications of *COL1A1* expression in macrophages, as well as its contribution to wound ECM and granulation tissue responses. This study does not address if these macrophages become collagen-producing myofibroblasts, retain their macrophage characteristics, or whether there is conversion from proinflammatory to anti-inflammatory phenotype. Moreover, in-depth in vivo studies are warranted to confirm whether these findings hold true in an actual wound environment. To date, there is a dearth of literature documenting the effects of CS-API or CSO on other cell types pertinent to the process of wound healing.

In addition to collagen subtypes, the study revealed enrichment of vimentin (*VIM*) and Actin alpha 2, smooth muscle (*ACTA2*), also known as alpha smooth muscle actin (α-SMA), expressing macrophages following CS-API treatment. Vimentin is an intermediate filament protein that primarily serves as a structural component of the cytoskeleton and plays important roles in wound healing^40^. Vimentin has been implicated in facilitating macrophage migration and host defenses during wound healing and tissue repair processes^40,41^. ACTA2 protein is primarily associated with smooth muscle cells. ACTA2 plays a crucial role in wound healing, contributing to structural and contractile functions of myofibroblasts^42^. ACTA2 and vimentin expressions have been reported in macrophages^41,43^. Various studies, including our own, have documented a pivotal role of vimentin and ACTA2 in transitioning macrophages into fibroblasts and myofibroblasts like cells such as seen in macrophage–myofibroblast transition (MMT)^44–48^. MMT was supported in vivo in the idiopathic pulmonary fibrosis (IPF) patients via single-cell RNA sequencing (scRNA seq) where approximately 50% of ACTA2^+^ cells also exhibited positivity for the macrophage marker CD68 suggesting presence MMT in vivo^49^. These findings provide additional support for a crucial role of ACTA2 in modifying the macrophages to fibroblast-like cells.

Clostridial collagenase has been shown to aid in the continued removal of immediate and accumulating necrotic tissue without affecting healthy tissue^5,50^. A unique feature of this collagenase is that it can cleave repeating Gly-X–Y collagen sequences at distinct Y-Gly bond harboring sites thus releasing collagen peptides^51^. Macrophages are known to internalize collagen by mannose receptors^52^. A study investigating underlying mechanisms of CS-API mediated cellular effects provided evidence that collagen-associated and other ECM-derived peptides are primarily responsible for its downstream angiogenic responses^11^. We have reported macrophage conversion to a pro-angiogenic and anti-inflammatory phenotype by collagen peptides^53^. This evidence along with the report that collagen peptides can induce TGFb1-smad pathway suggests that ECM peptides associated with CS-API treatment of wounds may be responsible for its pro-ECM effects on macrophages^54^. In our model involving isolated macrophages treated with collagenase in culture, it is acknowledged that extracellular collagen secreted from macrophages will be the only substrate for the enzyme for production of collagen peptides. Additional mechanisms of collagenase action on isolated macrophages on inducing the pro-ECM phenotype cannot be disregarded. Previous research from our group highlighted the activation of the PGE2-EP4 pathway after treating isolated macrophages with collagenase in culture (CSO)^12^. This pathway is recognized for its role in activating collagen gene expression^55^.

This study provides initial evidence that clinically approved wound debridement agent CSO has the potential to improve the wound ECM response by converting macrophages to an ECM supporting fibroblast-like phenotype. Our earlier report established the significance of the physiological conversion of wound-site macrophages to fibroblasts in granulation tissue, contributing to tissue remodeling and this response is compromised in chronic diabetic wounds^22^. The findings that CSO can augment the physiological conversion of macrophages to fibroblast-like cells hold immense significance. This clinical intervention, already utilized for wound care, can be readily repurposed to improve the extracellular matrix (ECM) response in chronic wounds. The studies have reported that CSO in combination with sharp debridement as opposed to sharp debridement alone provided better healing outcomes and suggest a role of CSO beyond debridement^29,56^. Further research is needed to fully understand the underlying mechanisms and functional implications of macrophage conversion and ECM remodeling in the context of CSO treatment. Such knowledge can pave the way for the development of targeted strategies to enhance wound healing outcomes and improve the quality of life for individuals with chronic wounds.

## Materials and methods

### Peripheral Blood Monocyte Derived Macrophages (MDMs) and CS-API treatment

Human monocyte-derived macrophages (MDM) were harvested from fresh blood leukocyte source packs (Versity, Indianapolis, Indiana) by density gradient centrifugation using a Ficoll-Hypaque density gradient (GE Healthcare, formerly Amersham Biosciences, Piscataway, NJ) as previously described^57^. Positive selection for monocytes was performed using CD14 antibody conjugated to magnetic beads (Miltenyi Biotec, Auburn, CA). Purity of these preparations of monocytes was > 90% as determined by fluorescence-activated cell sorting analyses using CD14 antibodies. The experiments were performed using MDM from multiple donors (n = 4–5). Differentiation of these cells to macrophages (MDMs) was performed as described^57^. The MDMs were seeded in 12-well plates at a density of 1.5 × 10^6^ and treated with the active ingredient of CSO, CS-API (250 ng/ml). The CS-API was dissolved in TESCA buffer (pH 7.4) and provided to cells as reported by us^12^. The control cells were treated with TESCA buffer (TES: N-Tris(hydroxymethyl)methyl-2-aminoethanesulfonic Acid); TES + CaCl_2_ (TESCA) buffer alone for periods specified in figure legends. The media and cells were collected for downstream molecular analysis.

### Single-cell RNA-sequencing

Single-cell suspensions were generated from MDM cells following 24 h of CS-API treatment. The viability of the cells was determined to be > 98% by performing cell counting and viability assays using propidium iodide (PI) staining and flowcytometry. PI is a membrane impermeant dye that is generally excluded from viable cells. The resulting cell suspension was utilized for scRNA-Seq using the 10 × Genomics platform using Chromium Next GEM Single Cell 3′ GEM, Library & Gel Bead kit v3.1and sequenced on an Illumina NovaSeq 6000 as reported by us^58–60^.

For data analysis the raw sequencing data by demultiplexing and trimming adapter sequences using the 10 × Genomics Cell Ranger software. The preprocessed data to a reference genome using read aligners such as STAR. The data was further processed using the Partek Flow^®^ software including quality control steps to remove low-quality cells based on the following criteria total reads, number of genes detected, and mitochondrial gene content. The data was normalized using counts per million (CPM). The principal component analysis (PCA) and t-distributed stochastic neighbor embedding (t-SNE) techniques were utilized to visualize and explore the cell population structure. The cell clusters were identified using graph-based approaches. Differentially expressed genes between clusters were determined using a D-Seq module with statistical tests. To determine the biological processes and pathways associated with specific cell clusters of MDMs, Ingenuity pathway analysis (IPA, Qiagen) was performed^58,59^.

### RT2 profiler PCR

Gene expression profiling on ECM-specific pathways was done using a 96-well human RT2 Profiler PCR Array (Qiagen, Catalog 330,231 PAHS-013ZA, MD, USA). The array comprised of 96 wells. Each well contained all the reagents required for the PCR reaction in addition to a primer for a single gene. MDM cells were treated with CS-API (250 ng/ml) or control (TESCA buffer). cDNA was transcribed using an RT2 First Strand Synthesis Kit (QIAGEN). The resulting cDNA was diluted and loaded into the predesigned RT2 Profiler PCR Array plates. mRNA expression levels were quantified employing the 2^(−ΔΔct)^ relative quantification method.

### RNA extraction, reverse transcription, and quantitative RT-PCR

mirVana RNA isolation kit (ThermoFisher, Waltham, MA) was used according to the manufacturer’s instructions to extract total RNA as previously described^22^. Quantification of mRNA was done by real-time or quantitative (Q)PCR assay using double-stranded DNA binding dye SYBR Green-I and primers specific for the *Col1A1* gene as described previously^12,61,62^. *18S* or *β-ACTIN* were used as reference housekeeping genes.

### Immunocytochemistry (ICC)

Cytospun MDM cell suspensions on glass slides were fixed in 4% paraformaldehyde for 10 min at room temperature. Following fixation, cells were washed with PBS, blocked in 10% normal goat serum (NGS) for 30 min, and were incubated in primary antibodies Col1A1 (1:200; NBP1-77458F; FITC tagged; Novus), CD68 (1:200; ab213363, Abcam). Fluorescence-tagged secondary antibody detection was performed with Alexa Fluor 568 secondary antibody (1:200, Life Technologies) as described previously^12^. The cells were counter stained by DAPI (nuclear stain, blue). Fluorescent images were collected using axioscan imager (Carl Zeiss, Germany). Image analysis was performed using Zen (Zeiss) software.

### ELISA

Levels of Col1A1 secreted by hMDMs in the presence or absence of CS-API were measured using commercially available human collagen type I, alpha 1 ELISA kit (MyBioSource, catalog# MBS703198, San Diego, CA) as per manufacturer’s instructions^12,53,62,63^.

### Data collection and statistical analyses

Data are reported as mean ± SEM of 4–8 experiments as indicated in respective figure legends. Student's t-test was used to determine significant differences between the means. *p* < 0.05 was considered statistically significant.

## Data Availability

The data that support the findings of this study are available on request from the corresponding author SR. The datasets generated and/or analyzed during the current study are available in the GEO repository. The following secure token has been created to allow review of record GSE243622 while it remains in private status.
